# Predictors of the unfavorable outcomes in acute ischemic stroke patients treated with alteplase, a multi-center randomized trial

**DOI:** 10.1038/s41598-024-56067-5

**Published:** 2024-03-12

**Authors:** Mohamed G. Zeinhom, Mohamed Fouad Elsayed Khalil, Islam Fathallah Mohamed Kamel, Ahmed Mohamed Kohail, Sherihan Rezk Ahmed, Ahmed Elbassiouny, Ashfaq Shuaib, Omar M Al-Nozha

**Affiliations:** 1 Neurology Department, Faculty of Medicine, Kafr El-Sheikh University, Elgeish Street, Kafr El-Sheikh, Egypt; 2 Neurology Department, Saudi German Hospital Madinah, Abyar Ali. Al Jamaat Rd., Madinah, Saudi Arabia; 3 Neurology Department, Faculty of Medicine, Tanta University, El-gash St., Tanta, Egypt; 4 Neurology Department, Faculty of Medicine, Al-Azhar University, ELmokhaim St., Cairo, Egypt; 5 Neurology Department, Faculty of Medicine, Ain Shams University, ELabbasia St., Cairo, Egypt; 6 Division of Neurology, Department of Medicine, University of Alberta, Clinical Sciences Building, Edmonton, AB T6G 2R3 Canada; 7 Medicine Department, College of Medicine, Taibah University, Janadah Bin Umayyah Rd., Tayba, Madinah Saudi Arabia

**Keywords:** Alteplase, Functional outcomes, Acute ischemic stroke, Egypt, Saudi Arabia, Neurology, Neurological disorders

## Abstract

Worldwide, stroke is a leading cause of long-term disability in adults. Alteplase is the only approved treatment for acute ischemic stroke (AIS) and results in an improvement in a third of treated patients. We evaluated the post-stroke unfavourable outcome predictors in alteplase-treated patients from Egypt and Saudi Arabia. We assessed the effect of different risk factors on AIS outcomes after alteplase in Egypt and Saudi Arabia. Our study included 592 AIS alteplase-treated patients. The relationship between risk factors, clinical presentation, and imaging features was evaluated to predict factors associated with poor outcomes. An mRS score of three or more was used to define poor outcomes. Poor outcome was seen in 136 patients (23%), and Patients with unfavourable effects had significantly higher admission hyperglycaemia, a higher percentage of diabetes mellitus, cardioembolic stroke, and a lower percentage of small vessel stroke. Patients with higher baseline NIHSS score (OR 1.39; 95% CI 1.12–1.71; P = 0.003), admission hyperglycaemia (OR 13.12; 95% CI 3.37–51.1; P < 0.001), and post-alteplase intracerebral haemorrhage (OR 7.41; 95% CI 1.69–32.43; P = 0.008) independently predicted unfavourable outcomes at three months. In AIS patients treated with alteplase, similar to reports from other regions, in patients from Egypt and Saudi Arabia also reveal that higher NIHSS, higher serum blood sugar, and post-alteplase intracerebral haemorrhage were the predictors of unfavourable outcomes three months after ischemic stroke.

Trial registration: (clinicaltrials.gov NCT06058884), retrospectively registered on 28/09/2023.

## Introduction

Stroke is a leading contributor to long-term disability in adults. It is the second leading cause of mortality on a global scale. Developing countries bear a disproportionate burden of stroke, representing 66% of the total stroke cases worldwide^1^.

The administration of alteplase within a time frame of up to four and a half hours following onset of symptoms has been shown to improve long-term outcomes in individuals suffering from stroke. Moreover, alteplase and tenectaplase remain the only approved medical therapeutic interventions for the management of acute ischemic stroke^2^.

In the context of an ischemic stroke, the mitigation of brain injury and impairment can be achieved by promptly restoring blood flow to the penumbra region prior to its complete transformation into infarcted tissue. Nevertheless, some variables such as the extent of pre-existing brain damage prior to the restoration of blood flow, changes in blood pressure during the administration of thrombolytic therapy, and an excessive amount of glucose during the reestablishment of blood flow, may potentially hinder recovery and adversely affect prognosis of a stroke^3^.

Some patients exhibited suboptimal outcomes following alteplase administration, this is potentially attributable in part to varying risk factors associated with the onset of ischemic stroke^4^.

Factors that contribute to prognosis following alteplase treatment are not very clear. Several studies have suggested that hypertension, blood sugar levels, hyperlipidemia, and heart disease do not serve as prognostic factors for functional results^5,6^.

Other studies report that hyperglycemia is associated with poor outcomes in anterior circulation large-vessel stroke, and still other studies report that dyslipidemia may be related to post-ischemic stroke poor outcomes^7,8^.

Given the ongoing discourse surrounding the efficacy of various risk factors for ischemic stroke in predicting poor post-alteplase outcomes, particularly within the Middle East, our study sought to assess the predictors of unfavorable clinical outcomes in Egyptian and Saudi patients who have experienced an ischemic stroke and subsequently received alteplase.

## Methods

### Trial design

We conducted our open-label prospective cohort study between January 2022 and October 2023 after approval of the ethical committee of the faculty of medicine at Kafr el-sheik University, and Nasr city insurance hospital in Egypt, the ethical committee of the Saudi German hospital in Madinah, Saudi Arabia.

1184 patients who presented with first-ever AIS and were eligible for receiving alteplase underwent randomization and our study included 592 patients who met our criteria.

Our study adheres to CONSORT guidelines and includes a completed CONSORT checklist as an additional file.

### Participants

Our study included 592 AIS patients treated with alteplase within four and half hours of which 444 patients were recruited from Kafr el-Sheikh hospital, and Nasr city insurance hospital in Egypt, and 148 patients were recruited from Saudi German hospital in Madinah, Saudi Arabia, and all patients included in our study met our inclusion criteria.

The study consisted of two distinct groups. The first group consisted of 456 patients who experienced favourable outcomes, while the second group comprised 136 patients who experienced unfavourable outcomes.

### Eligibility criteria

We enrolled individuals of both genders, aged between 18 and 75 years, who presented with acute first-ever ischemic stroke and were eligible for thrombolysis. The diagnosis was confirmed based on a thorough clinical assessment, including a detailed medical history, physical examination, and specific brain imaging results. Patients with previous transient ischemic attacks who had alteplase contraindications were excluded from the study. We included patients with NIHSS ≥ 3 and less than 25^9^.

We excluded patients who had not been followed up on for 90 days after enrollment, those with alteplase contraindications, or did not receive the total dose of alteplase due to any reason, patients with a known history of persistent or recurrent CNS pathology (e.g., epilepsy, meningioma, multiple sclerosis, history of head trauma with a residual neurological deficit) and patients who had recurrent ischemic stroke diagnosed by appropriate clinical history and/or MRI brain findings were also not included.

We excluded patients with symptoms of major organ failure, active malignancies, or an acute myocardial infarction within the previous 6 weeks.

We also excluded pregnant and lactating patients, those with stroke due to venous thrombosis and stroke following cardiac arrest.

### Interventions

The data collection encompassed many demographic and clinical variables, including age, gender, medical history pertaining to hypertension (HTN), ischemic heart disease (IHD), hyperlipidemia, diabetes mellitus, tobacco use, and the duration between symptoms' onset and treatment initiation. The diagnosis of ischemic stroke was established through a comprehensive evaluation that included a detailed clinical history and examination and the utilization of CT brain and MRI brain using stroke protocol: T1W, T2W, FLAIR, DWI, T2 Echo Gradient*,* CTA, or MRA if CTA was contraindicated, from the aortic arch through the circle of Willis. Two neuroradiologists reviewed C.T. and MRI source images. Cerebrovascular vessels were divided into segments: supra-clinoid internal carotid artery, first-division middle cerebral artery (M1), second-division middle cerebral artery (M2), first-division anterior cerebral artery (A1), second-division anterior cerebral artery (A2), basilar artery (B.A.), intracranial vertebral artery (V.A.), first division posterior cerebral artery P1), and second division posterior cerebral artery (P2). A neuroradiologist determined whether any of these vascular segments were occluded. If there was no vascular occlusion, the patient was documented as having no large vessel occlusion. If one or more vascular segments were occluded and the patient was eligible for endovascular management, pre-stroke mRS score of 0–1; (2) causative occlusion of the internal carotid artery or MCA segment 1 (M1); (3) age ≥ 18 years; (4) NIHSS score of ≥ 6; (5) ASPECTS of ≥ 6; and (6) treatment can be initiated (groin puncture) within 6–16 h of symptom onset. Then the procedure was done by a senior neuro-intervention consultant using Philips Biplane Allura Xper FD20/15 release 8.2 with X-ray generator 100 kva, and the procedure was performed under general anesthesia in an angiography suite with biplane digital subtraction and road-mapping capabilities.

We assessed the baseline Alberta Stroke Program Early CT score (ASPECTS) of all patients included in our study, which is a 10-point quantitative topographic CT scan score used in patients with middle cerebral artery (MCA) stroke. A segmental assessment of the MCA vascular territory is made, and 1 point is deducted from the initial score of 10 for every region involved: caudate, putamen, internal capsule, and insular cortex; in posterior circulation stroke, we used pc-ASPECTS which is a 10 point scale, where points are lost for each region affected, thalami (1 point each), occipital lobes (1 point each), midbrain (2 points), pons (2 points), and cerebellar hemispheres (1 point each)^10^.

All the patients underwent a series of diagnostic tests, including transoesophageal echocardiography, 12-lead routine ECG and 24-h of continuous cardiac rhythm monitoring, carotid duplex, and blood pressure assessment, and we diagnosed hypertension when systolic blood pressure was more than 130 mmHg and/or diastolic blood pressure was more than 85 mm/Hg in at least three different occasions^11^, renal function, liver functions, coagulation profile, complete blood count, fasting, postprandial blood sugar, and HbA1C.

We diagnosed diabetes when fasting plasma glucose level was more than 126 mg/dL and/or casual plasma glucose was more than 200 mg/dL and/or HbA1C was more than 6.5^11^, and we diagnosed admission hyperglycemia when admission blood glucose value was more than 140 mg/dL^7^. Regarding the management of hyperglycemia, we aimed to maintain blood glucose levels below 140 mg/dL and 180 mg/dL, so we withhold all usual antidiabetic treatments and use periodic subcutaneous regular insulin injections, adjusted according to blood glucose levels. Patients received rapid-acting insulins immediately after meals based on the amount of carbohydrates consumed. For standard meals containing 60 g of carbohydrates, four units of rapid-acting analogue insulin were used, and blood glucose level was followed up every three hours. If the blood glucose concentration did not reach the target at 24 and 48 h, the subcutaneous insulin dose was increased, including long-acting basal insulin^10^.

We diagnosed hyperlipidemia when blood cholesterol was more than 200 mg/dL, triglycerides were more than 150 mg/dL, LDL-cholesterol was more than 100 mg/dL and/or HDL-cholesterol was less than 40 mg/dL). ^12^ Regarding the management of hyperlipidemia, patients with LDL-C > 100 mg/dL and without ischemic heart disease, cardiac sources of embolism, intracranial, or carotid atherosclerosis received daily 80 mg of atorvastatin. In contrast, patients with LDL-C > 100 mg/dL and who had atherosclerotic disease (intracranial, carotid, aortic*,* or coronary) received 80 mg daily of atorvastatin and 10 mg of ezetimibe. The patients received antihyperlipidemic treatment to achieve LDL-C < 70 mg/dL and performed follow-up fasting lipid profile after 4–12 weeks of starting antihyperlipidemic agents^13^.

All patients underwent CT imaging on admission and another CT after 36–48 h to assess the occurrence of hemorrhagic transformation.

In accordance with the guidelines set forth by the American Heart Association/American Stroke Association (AHA/ASA), inclusion, and exclusion criteria for alteplase were established; 0.9 mg/kg of alteplase up to a maximum dose of 90 mg was administered intravenously to eligible individuals within 4.5 h of the beginning of their clinical manifestations (10% bolus, 90% infusion in 1 h). After receiving IV-alteplase, all patients continued their management and rehabilitation in the stroke unit.

If any patient had neurological worsening (National Institutes of Health Stroke Scale [NIHSS] increased by more than 4 points)^7^, we performed an additional CT scan to exclude symptomatic intracranial hemorrhage.

Hemorrhagic transformation was classified according to the European cooperative acute stroke study (ECASS) classification^14^.

Using the PLATO bleeding definition, we estimated the hemorrhagic complications assessed^15^.

We evaluated all the patients on admission and after 24 h by assessing the NIHSS, and we showed a decrease of four points or more in the NIHSS score as a significant improvement^16,17^ Moreover, we considered hemorrhagic transformation symptomatic if the NIHSS score increased by 4 points or more^7^.

As for hemorrhagic transformation treatment and after performing follow-up CT brain, CBC, PT, (INR), aPTT, fibrinogen level, and type and cross-match, patients received Cryoprecipitate (includes factor VIII): 10 U infused over 10–30 min and an additional dose for fibrinogen level of < 200 mg/dL^9^, or fresh frozen plasma at rate of 12 ml/kg if cryoprecipitate was not available^13^ and Supportive therapy, including blood pressure management, intracranial pressure management using dehydrating measures, control of temperature, and glucose levels.

As for other complications as orolingual Angioedema associated with alteplase, we maintained airway and endotracheal intubation was considered if oedema involving larynx, palate, floor of mouth, or oropharynx with rapid progression (within 30 min), we stopped alteplase infusion, patient received IV methylprednisolone 125 mg, IV diphenhydramine 50 mg, ranitidine 50 mg IV, and if there was further increase in angioedema, administer epinephrine (0.1%) 0.3 mL subcutaneously or by nebulizer 0.5 mL^13^, patients who developed seizures, were managed by performing maintaining vital signs, receiving appropriate antiepileptic medications and had CT and/or MRI brain and 30-min electroencephalography (EEG) and were classified into early-onset seizures (i.e., if seizures developed during the first 7 days after stroke), late onset-seizures (i.e., if seizures started after the first week), and post-stroke epilepsy, and post-stroke epilepsy was considered if patients had recurrent seizures, showed seizures after attempts to withdraw antiepileptic drugs, or if patients were maintained in treatment with anti-epileptic drugs because of a great chance of seizure recurrence as evaluated by EEGs, neuroimaging findings, and physician evaluation^18^.

We assessed mRS after 90 days via a 10-min telephone interview. With patients or their primary caregivers to detect the score, all of our patients had baseline mRS of zero. mRS two or less was considered a favorable outcome^2,19^.

### Outcomes

We had two primary outcomes. The first was to detect the modified Rankin scale (mRS) score after 90 days via a 10-min telephone interview with patients or their primary caregivers. mRS two or less was considered a favorable outcome^20^.

The second was to detect the predictors of unfavorable outcomes after 90 days of AIS by the means of multivariate Logistic regression analysis.

#### Sample size

We employed G-power software to calculate the power of our sample size, which was 95%, given a two-sided confidence level of 95% and an alpha error of 5%.

#### Randomization

We employed a web-based centralized blocked randomization plan to allocate patients in a one-to-one ratio to be involved in our study or not.

Before randomization, we got formal written informed consent from all eligible patients or their first order of kin.

### Statistical analysis of the data

We used the IBM SPSS software package, version 20.0 (Armonk, NY: IBM Corp.), to analyze our data and base all efficacy analyses on the per-protocol principle. Both the primary and secondary outcomes underwent separate statistical analyses. Depending on their distribution, as determined by the Shapiro–Wilk test, we described numerical data as means S.D. or median and interquartile range (IQR). We also reported categorical data using numbers and percentages. The Mann–Whitney U test was used to compare the irregularly distributed numerical data, while Pearson's chi-square was utilized to correlate categorical data. In our study, there were all the data.

### Ethical approval

Our study had the approval of the ethical committee of Kafr-ELsheikh university, and the ethical reference number is (KFSIRB200-66). All methods of our trial were performed in accordance with the guidelines and regulations of faculty of medicine Kafr-ELsheikh university (FMASU) which is organized and operated according to the guidelines of international council on harmonization (ICH) Anesthesiology and the Islamic organization for medical service (IOMS), the united states office for human research protections, and the united states code of federal regulations and operates under federal wide assurance no (FWA00001785). Trial registration: We registered our trial retrospectively on ClinicalTrials.gov, "Predictors of AIS Unfavorable Outcomes" (NCT06058884)—28/09/2023.

### Consent to participate

Before randomization, formal written informed consent was obtained from the included patients or their first-degree relatives.

## Results

**Figure 1:**
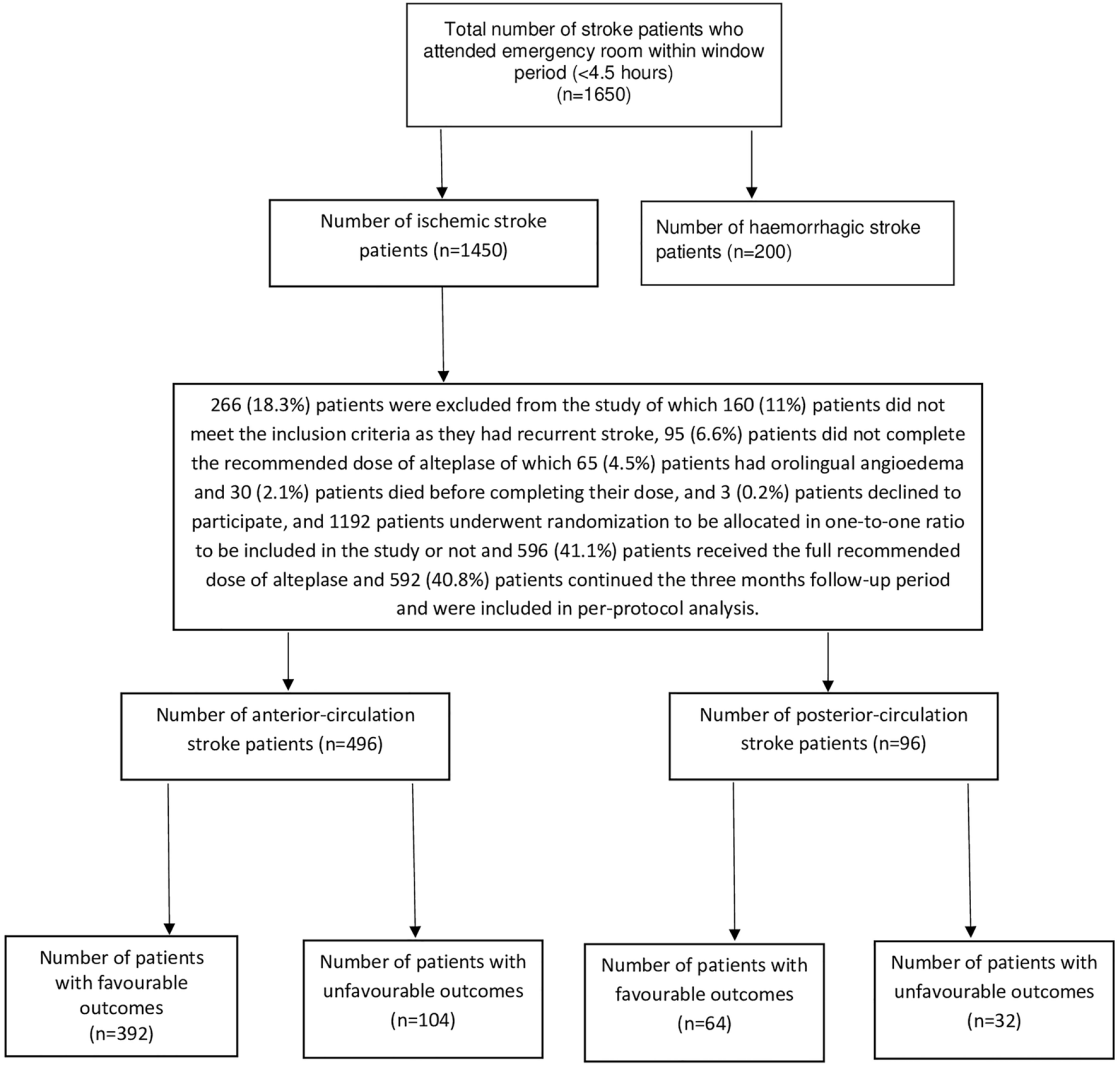
Study flow diagram.

Overall, a cohort of 1192 patients underwent randomization. Out of these patients, 592 individuals (220 females and 372 males) were randomly assigned to be included in our study, received the total recommended dose of alteplase, completed the study during the three-month follow-up period, and 456 patients (77%) had favorable outcomes (mRS equal or less than 2 points), 224 patients (37.8%) had excellent outcome (mRS equal or less than 1 point) however 136 patients (23%) had unfavorable outcomes (mRS more than 2 points) as shown in Fig. 1.

There were no significant differences between the two groups related to baseline characters apart from baseline NIHSS, which was statistically significantly higher in patients with unfavorable outcomes with a P value 0.002, as shown in Table 1.

**Table 1:** Comparison between patients' different baseline characters.

Demographic data	All patients (n = 592)	Unfavorable outcome (n = 136)	Favorable outcome (n = 456)	Test of significance	P value
Male, no, (percentage)	373.0 (63.0%)	93.0 (68.4%)	280.0 (61.4%)	*Χ*^2^ = 2.189	0.140
Age (years), Median (IQR)	58.0 (53.0–66.0)	59.0 (53.0–67.0)	58.0 (53.0–66.0)	U = 32,220	0.490
Anterior circulation, no, (percentage)	496.0 (83.8%)	112.0 (82.4%)	384.0 (84.2%)	*Χ*^2^ = 0.266	0.610
Posterior circulation, no., (percentage)	96.0 (16.2%)	24.0 (17.6%)	72.0 (15.8%)		
NIHSS at time of admission Median (IQR)	11.0 (9.0–18.0)	13.5 (9.0–21.0)	11.0 (9.0–14.0)	U = 36,360*	0.002*
Door to needle time (min.) Median (IQR)	60.0 (54.3–65.0)	60.0 (55.0–66.0)	60.0 (54.0–69.0)	U = 29,200	0.590
Time of receiving IV rtPA from stroke onset (min.) Median (IQR)	174.0 (164.8–187.0)	174.0 (163.3–188.5)	174 (172.0–187.0)	U = 30,970	0.980
ASPECTs score, Median (IQR)	5.0 (4.0–7.0)	5.0 (4.0–7.0)	5.0 (4.0–7.0)	U = 29,814	0.49
Patients who developed orolingual angioedema, no., (percentage)	12.0 (2.0%)	4.0 (2.9%)	8.0 (1.8%)	*Χ*^2^ = 0.743	0.39
Patients who developed seizures, no., (percentage)	10 (1.7%)	3.0 (2.2%)	7.0 (1.5%)	*Χ*^2^ = 0.284	0.59

*SD* standard deviation, *χ*^2^ Chi square test, *U* Mann Whitney test, *p* p value for comparing between unfavorable and favorable.

*Statistically significant at p ≤ 0.05.

Regarding the analysis of the different risk factors, there was no significant difference between the two groups regarding hyperlipidemia, hypertension, ischemic heart disease, or smocking; in addition, there was no significant difference between the two groups regarding treatment modalities other than alteplase, including endovascular management, cryoprecipitate, fresh frozen plasma or antiepileptic medication. Patients with unfavorable outcomes had a significantly higher percentage of atrial fibrillation, higher admission hyperglycemia, and a higher percentage of diabetes millets when compared to Patients with favorable outcomes with P values as follows < 0.001, 0.001, 0.013, respectively, as shown in Table 2.

**Table 2:** Comparison between the patients' risk factors and different management.

	All patients (n = 592)	Unfavorable outcome (n = 136)	Favorable outcome (n = 456)	Test of significance	P value
Risk factor, no, (percentage)					
Hyperlipidemia	212.0 (35.8%)	40.0 (29.4%)	172.0 (37.7%)	χ^2^ = 3.145	0.076
Diabetes mellitus	293.0 (49.5%)	80.0 (58.8%)	213.0 (46.7%)	χ^2^ = 6.149^*^	0.013*
Admission hyperglycemia	198.0 (33.4%)	61.0 (44.9%)	137.0 (30.0%)	χ^2^ = 10.321^*^	0.001*
Hypertension	404.0 (68.2%)	100.0 (73.5%)	304.0 (66.7%)	χ^2^ = 2.277	0.130
Atrial fibrillation	156.0 (26.4%)	52 .0 (38.2%)	104.0 (22.8%)	χ^2^ = 12.848^*^	< 0.001*
Ischemic heart disease	66.0 (11.1%)	16.0 (11.8%)	50.0 (11.0%)	χ^2^ = 0.068	0.795
Smoking	196.0 (32.3%)	47.0 (34.6%)	144.0 (31.6%)	χ^2^ = 0.426	0.514
Etiology, no, (percentage)					
Large artery atherosclerosis	107.0 (18.1%)	32.0 (23.5%)	75.0 (16.4%)	χ^2^ = 3.548	0.060
Cardio-embolism	146.0 (24.7%)	48.0 (35.3%)	98.0 (21.5%)	χ^2^ = 10.748^*^	0.001*
Small artery occlusion	176.0 (29.7%)	24.0 (17.6%)	152.0 (33.3%)	χ^2^ = 12.388^*^	< 0.001*
Stroke of other determined etiology	163.0 (27.5%)	32.0 (23.5%)	131.0 (28.7%)	χ^2^ = 1.419	0.234
Management other than alteplase no, (percentage)					
Endovascular management	30.0 (5.1%)	6.0 (4.4%)	24.0 (5.3%)	χ^2^ = 0.158	0.69
Cryoprecipitate or Fresh frozen plasma	52.0 (8.8%)	10 (7.4%)	42 (9.2%)	χ^2^ = 0.451	0.50

*χ*^2^ Chi square test, *U* Mann Whitney test, *p* p value for comparing between unfavorable and favorable.

*Statistically significant at p ≤ 0.05.

Patients with unfavorable outcomes had a statistically significant higher percentage of cardioembolic stroke and a lower percentage of small-vessel disease stroke with P values 0.001 and < 0.001, respectively, as shown in Table 2.

We evaluated the relative contribution of the different variables for poor outcomes (mRS score was more than 2 points) at three months. We found that some factors had statistically significant relations with unfavorable outcomes as follows, baseline NIHSS on admission (P < 0.001), admission hyperglycemia (P = 0.002), post-alteplase intracerebral hemorrhage (P < 0.001), diabetes mellitus before treatment (P = 0.015), cardioembolic stroke (P = 0.002). Multivariate regression model revealed that only baseline NIHSS score (odds ratio [OR], 1.39; 95% CI 1.12–1.71; P = 0.003), admission hyperglycemia (OR 13.12; 95% CI 3.37–51.1; P < 0.001), and post-alteplase intracerebral hemorrhage (OR 7.41; 95% CI 1.69–32.43; P = 0.008) independently predicted poor outcome (mRS, more than 2 points) at three months, as shown in Table 3.

**Table 3:** Univariate and multivariate Logistic regression analysis for the different risk factors of unfavorable outcomes (n = 136).

	P	Univariate OR (LL–UL 95% CI)	p	^#^Multivariate OR (LL–UL 95% CI)
Male	0.509	1.314 (0.584–2.959)		
Age at time of presentation	0.850	1.004 (0.968–1.041)		
Lesion location (Posterior circulation)	0.192	1.885 (0.727–4.885)		
ASPECT score	0.57	0.921 (0.981–1.32)		
NIHSS at time of admission	< 0.001*	1.367 (1.202–1.555)	0.003*	1.388 (1.120–1.719)
Admission hyperglycemia	0.002*	3.711 (1.616–8.521)	< 0.001*	13.105 (3.364–51.057)
Door to needle time (min.)	0.564	0.992 (0.967–1.018)		
Time of receiving IV rtPA from stroke onset (min)	0.505	1.003 (0.994–1.013)		
Post rtPA intracerebral hemorrhage	< 0.001*	15.0 (4.426–50.842)	0.008*	7.410 (1.693–32.431)
Hyperlipidemia	0.069	0.428 (0.172–1.067)		
Diabetes mellitus	0.015*	2.643 (1.207–5.788)	0.104	2.505 (0.827–7.583)
Hypertension	0.104	2.020 (0.865–4.716)		
CAD	0.838	1.133 (0.340–3.773)		
Atrial fibrillation	0.002*	4.238 (1.706–10.529)	0.068	3.396 (0.915–12.595)
Smoking	0.470	1.341 (0.605–2.975)		
Large artery atherosclerosis	0.055	2.281 (0.982–5.301)		
Cardio-embolism	0.002*	4.238 (1.706–10.529)	0.068	3.396 (0.915–12.595)
Small artery occlusion	0.003*	0.103 (0.024–0.452)	0.625	0.587 (0.070–4.949)
Stroke of undetermined etiology	0.227	0.549 (0.208–1.451)		
Orolingual angioedema	0.42	0.841 (0.811–1.12)		
Seizures	0.38	0.742 (0.694–0.982)		
Endovascular management	0.52	0.912 (0.857–1.14)		
Fresh frozen plasma	0.74	1.07 (0.918–1.12)		

*OR* odd`s ratio, *rtPA* recombinant tissue plasminogen activator, *CI* confidence interval, *LL* lower limit, *UL* upper limit, *CAD* coronary artery disease.

^#^All variables with p < 0.05 was included in the multivariate.

*Statistically significant at p ≤ 0.05.

## Discussion

Ischemic strokes may be attributed to embolic or thrombotic occlusions of intracranial or extracranial vessels. Spontaneous partial recanalization of the occluded cerebral vessels might occur only in less than 20% of patients during the first day after stroke onset^21–23^.

Alteplase is a fibrinolytic agent that converts plasminogen to the proteolytic enzyme plasmin, which lyses fibrin as well as fibrinogen, leading to clot lysis and reopening occluded vessels^24^.

Thrombolysis using alteplase recanalizes the occluded arteries quickly, restores perfusion to the ischemic penumbra and limits the consequences of cerebral ischemia^22^.

Alteplase has some complications related to reperfusion injury (interaction between blood and injured tissue) in the postischemic stage, extending the tissue damage, and this injury is influenced by individual differences of the reperfusion window, the duration and severity of ischemia and the collateral circulation^22,25^.

Few studies evaluated the predictors of poor outcomes following alteplase in AIS patients in Egypt and Saudi Arabia. Still, all of them were either retrospective, non-randomized or non-registered studies, which limited the validity and generalizability of their results^26–30^.

We conducted our prospective study as the first randomized clinical trial that evaluated the potential predictors of unfavorable clinical outcomes in AIS patients who received alteplase in Egypt and Saudi Arabia, aiming to improve the evaluation of ischemic stroke prognosis and enhance and maintain stroke services in the Middle East.

In our study, 23% of the patients had poor clinical outcomes (mRS more than 2), and this agrees partially with the findings of Kenmuir and colleagues., 2015, and Papamichalis and colleagues., 2018 who found that 35% and 33%, respectively, of AIS patients who were treated with alteplase had poor outcomes^31,32^. Our results are at odds with Eldeeb et al., and Mehrpour et al. who found that 44% and 49% of acute ischemic AIS patients treated with alteplase had poor outcomes^4,30^. The differences may be related to the type and severity of strokes enrolled in the various studies.

Our good results may be attributed to the shorter door-to-needle time in our study (53.6 min ± 15.3) and the higher percentage (32%) of small vessel ischemic stroke patients included in our study.

Regarding the analysis of the baseline characters of the patients, we found that age, door to needle time, gender, baseline ASPECT score, and occurrence of orolingual angioedema or seizures were not predictors of poor outcomes in AIS patients treated with alteplase; our findings were in agreement with the findings of Tai and colleagues., 2019, who found that age was not a predictor of functional outcome in AIS patients treated with alteplase based on mRS after three months with P value 0.66^33^, and the findings of Eldeeb et al.^30^, who stated that door-to-needle time was not associated with poor outcomes regarding mRS after six months with P value 0.15, and the findings of the third international stroke trial (IST-3) who found that no imaging findings predicted the effect of alteplase on functional independence or symptomatic intracranial hemorrhage^34^, and the findings of Mysilimi et al., who found that orolingual angioedema did not predict the alteplase outcomes^35^, and disagree with the findings of Thatikonda et al., who found that female gender was a predictor of poor function outcome in AIS patients treated with alteplase based on Barthel index on discharge with P value 0.02^36^ and the findings of Xu et al., 2016 who found that post-ischemic stroke seizures indicate poorer prognosis OR 1.64 (95% CI 1.32–2.02)^37^.

This difference may be ascribed to disparities in the study design, the genetic composition of the patients enrolled in our trial, or the divergent risk factors present within our study sample.

We found that mechanical thrombectomy was not a predictor of outcomes in AIS patients treated with alteplase; our findings disagreed with the findings of Bhatia and colleagues., 2023 who found that mechanical thrombectomy resulted in better clinical outcomes than conservative management for acute large vessel occlusion stroke^38^ and this could be explained by disparities in the study population as Bhatia included only patients aged 2–years old.

We found that higher baseline NIHSS was associated with poor functional outcomes; this agrees with Aoki et al., 2013 who found that higher baseline NIHSS was associated with poor functional outcomes regarding mRS after 3 months with P value 0.002, and Elsayed et al., 2019 who found that higher baseline NIHSS was associated with poor functional outcomes regarding mRS after three months with P value 0.001^26,39^. Furthermore, this could be explained as higher NIHSS being associated with more extensive cerebral infarction, which is often associated with substantial brain edema; this edema compresses the peripheral vasculature, extending the brain tissue ischemia; in addition, the hypoxia and cellular damage products increase the permeability of the vascular wall increasing the chances of hemorrhagic transformation after the release of the brain edema leading to poor functional outcomes^40^.

In our study, we found that patients with poor outcomes had higher admission hyperglycemia, a higher percentage of diabetes millets, post-alteplase intracerebral hemorrhage, and atrial fibrillation (AF); this agrees with the findings of Paciaroni et al., 2008 who found that post-alteplase intracerebral hemorrhage, atrial fibrillation, and higher blood glucose level were related to poor outcomes in AIS patients regarding mRS after three months (OR 12.20, 95% CI 5.58–26.67), (OR 5.25; 95% CI 2.27–12.14), and (OR 1.01; 95% CI 1.00–1.01), respectively^41^.

Concerning the analysis of the relationship between different ischemic stroke etiologies and the functional outcomes, we found that patients with poor outcomes had more frequent cardioembolic strokes and less frequent small vessel strokes; this agrees with the findings of Paciaroni et al., 2008, who found atrial fibrillation was related to poor outcomes in AIS patients regarding mRS after three months (OR 5.25; 95% CI 2.27–12.14), and Tai and colleagues., 2019, who found atrial fibrillation was related to poor outcomes in AIS patients regarding mRS after three months with P value 0.049^33,41^. We also found that hypertension, history of smoking, and hyperlipidemia were not associated with poor outcomes in AIS patients; these findings are in line with the findings of Tai and colleagues., 2019 who found that hypertension, history of smoking, and hyperlipidemia were not associated with poor outcomes in AIS patients regarding mRS after three months with P value 0.67, 0.45, 0.16, respectively^33^.

After stratifying the baseline characters, risk factors, and etiologies of our patients by multivariate analysis, we found that only baseline NIHSS, admission hyperglycemia, and post-alteplase intracerebral hemorrhage were the independent predictors of poor clinical outcomes in AIS who received alteplase and this agrees with the findings of Paciaroni et al., 2008. who found that post-alteplase intracerebral hemorrhage, atrial fibrillation, and higher blood glucose level were related to poor outcomes in AIS patients regarding mRS after three months (OR 12.20, 95% CI 5.58–26.67), (OR 5.25; 95% CI 2.27–12.14), and (OR 1.01; 95% CI 1.00–1.01), respectively^41^.

Despite the promising outcomes observed in our study, it is important to acknowledge certain constraints inherent in our study. Firstly, the prospective design employed in our research resulted in a small sample size of patients. Secondly, an additional avenue for improvement would have been the inclusion of patients from diverse Middle Eastern nations.

## Conclusion

In AIS patients treated with alteplase, similar to reports from other regions, in patients from Egypt and Saudi Arabia also reveal that higher NIHSS, higher serum blood sugar, and post-alteplase intracerebral haemorrhage were the predictors of unfavourable outcomes three months after ischemic stroke.

## Data Availability

The datasets generated and analyzed during the current study are not publicly available due to the ethical regulations of our university, but are available from the corresponding author (Mohamed G. Zeinhom) on reasonable request.

## Abbreviations

AIS: Acute ischemic stroke
CNS: Central nervous system
CT: Computerized tomography
ECASS: European cooperative acute stroke study
ECG: Electrocardiogram
FDA: Food and Drug Administration
INR: International normalization ratio
IQR: Inter quartile range
MRI: Magnetic resonance imaging
mRS: Modified Rankin scale
NIHSS: National institute of health stroke score
TPA: Recombinant tissue plasminogen activator
TIA: Transient ischemic attack
